# Peripheral blood soluble elastin and elastase as auxiliary diagnostic indicators for coronary artery ectasia

**DOI:** 10.3906/sag-1911-16

**Published:** 2021-06-28

**Authors:** Ruifeng LIU, Qianqian SHENG, Siwen LIANG, Huiqiang ZHAO

**Affiliations:** 1 Department of Cardiology, Beijing Friendship Hospital, Capital Medical University, Beijing China

**Keywords:** Coronary artery ectasia, soluble elastin, elastase, biomarker

## Abstract

**Background/aim:**

Damage to elastin fibres in coronary media might lead to coronary artery ectasia (CAE). This study evaluated whether CAE can be distinguished by detecting circulating soluble elastin (s-elastin), which is a degradation product of elastin fibres, and elastase, which is the main enzyme of elastin fibres.

**Materials and methods:**

Fifty-eight patients with CAE, 58 with coronary heart disease (CHD) and 61 with relatively normal coronary arteries, were included. Circulating s-elastin and elastase were measured, and receiver operating characteristic curves were used to demonstrate their respective optimal cut-off values for predicting CAE.

**Results:**

The concentrations of s-elastin and elastase were higher in the CAE group than in the CHD and relatively-normal-coronary groups. Their cut-off values for screening of CAE were 13.148 ng/mL and 25.549 ng/mL, respectively; for sensitivity of CAE were 0.690 and 0.773, respectively; and for specificity of CAE were 0.862 and 0.571, respectively. A combination of s-elastin and elastase in series (one of the two higher than its cut-off value) had a better sensitivity for screening for CAE, whereas their combination in parallel (both higher than their cut-off values) had a better specificity.

**Conclusion:**

Circulating s-elastin and elastase are promising biomarkers for assisting in CAE diagnosis.

## 1. Introduction

Coronary artery ectasia (CAE) refers to the limited or diffused dilatation of the coronary lumen 1.5 times larger than the normal range [1]. Its prevalence in individuals who underwent coronary angiography (CAG) is 1.2%–4.9%, and more than 80% of patients with CAE have concomitant coronary heart disease (CHD) [1,2]. CAE is mainly characterised by myocardial ischaemic changes and atypical angina pectoris; it could cause malignant cardiovascular events, such as myocardial infarction, coronary rupture, and sudden death [3]. The main pathological change in coronary dilation is the significant destruction of the extracellular matrix (ECM), particularly the defect of elastic fibres in the middle coronary artery [4,5]. Proteinases might participate in ECM destruction [6,7], especially elastase, which degrades elastin fibres into soluble elastin (s-elastin) [8,9]. Thus, theoretically, CAE may be screened by detecting peripheral elastase and s-elastin concentrations in addition to CAG and coronary computed tomographic angiography. Therefore, this study conducted a preliminary evaluation of s-elastin and elastase concentrations with respect to the auxiliary diagnosis of CAE.

## 2. Methods

This retrospective, case-control study was approved by the Ethics Committee of the Beijing Friendship Hospital and was in line with the Helsinki Declaration. All patients signed the informed consent form. The patients had undergone CAG at Beijing Friendship Hospital from 2015 to 2017 through either femoral or radial artery. The images were reviewed by two independent cardiologists. For this research, CAE was defined as the diameter of the dilated artery exceeding 1.5 times the diameter of the adjacent normal segment [10]. CHD (control group 1) was defined as ≥50% stenosis of ≥1 major coronary artery [11]. The degree of coronary artery stenosis was <20% in the relatively-normal-coronary group (control group 2). The medical records of all patients were detailed and complete; blood samples were collected after coronary angiography, separated immediately, and refrigerated at −80 °C within 6 h of collection.

For selecting patients with CAE, the following exclusion criteria were applied: acute coronary syndrome, cardiomyopathy, valvular heart disease, heart failure, collagen tissue disease, vasculitis, syphilis, chronic obstructive pulmonary disease, pulmonary hypertension, asthma, inflammatory bowel disease, early menopause, organic liver disease, chronic alcoholism, steroid or antiinflammatory drugs, kidney failure, or cancer within the last 3 months. Thus, 58 patients with CAE were selected. Furthermore, 58 patients with CHD and 61 relatively-normal-coronary patients (from the same period corresponding to 2015–2017) were randomly selected as the control group. According to the Markis classification method [5], patients with CAE were divided into four groups based on the extent of coronary involvement: type I — diffuse ectasia of two or three vessels, type II — diffuse disease in one vessel and localised disease in another vessel, type III — diffuse ectasia of one vessel only, and type IV — localised or segmental ectasia.

Circulating s-elastin and elastase were detected using enzyme-linked immunosorbent assay, according to the manufacturer’s instructions (Wuhan Elabscience Biotechnology, Wuhan, Hubei, China). First, the sandwich double-antibody principle was used, and 100 µL of serum samples were placed into the precoated anti-s-elastin and anti-elastase antibody microplates. These were incubated at 37 °C for 90 min. The plates were washed, the horseradish oxidase-labelled secondary antibody was added, and the samples were again incubated for 2 h at 37 °C. Next, the plates were washed again, the reaction substrate (TMB) was added, and the samples were incubated at 37 °C for 30 min. Glacial acetic acid was used to terminate the reaction; then, the absorbance was read at 450-nm wavelength on a microplate reader. The final concentrations were calculated from the corresponding standard curve.

SPSS v25.0 (IBM, Armonk, NY, USA) was used for all statistical analyses. All data were initially analysed using the Shapiro–Wilk test to assess for normality. Continuous data are presented as mean ± SD when normally distributed or median with interquartile range (IQR) when non-Gaussian in distribution. For normally or nonnormally distributed continuous data, one-way analysis of variance or Kruskal–Wallis test was used for the multigroup analyses, and further, Tukey’s HSD method or Dunnett tests were employed for the pairwise analysis, respectively. Nonparametric data were expressed as a percentage (%) using a chi-squared test between groups. The receiver operating curve (ROC) was used to describe the specificity and sensitivity distribution, and optimal cut-off values for s-elastin and elastase were selected for diagnosing coronary dilatation. The statistical test standard was α < 0.05.

## 3. Results

Table 1 shows that no significant differences were observed in most baseline characteristics, including age, sex, hypertension prevalence, blood lipids, liver and kidney functions, or other cardiovascular risk factors among the three groups (all P > 0.05); significant differences were observed only for family history of CHD and diabetes prevalence.

**Table 1:** Baseline characteristics for enrolled patients

	CAE (n = 58)	CHD (n = 58)	Relatively normal* (n = 61)	P
Age (years)	64.00 (58.75–73.25)	63 (55.75–69.25)	59.00 (56.00–68.00)	0.086
Sex, male (%)	35 (60.34%)	37 (63.79%)	36 (59.02%)	0.860
CHD family history, n (%)	16 (27.59%)	21 (36.21%)	8 (13.11%)	0.014a
Diabetes family history, n (%)	5 (8.62%)	10 (17.24%)	8 (13.11%)	0.403
Heart rate (beats/min)	70.57 ± 10.12	70.93 ± 10.07	72.26 ± 10.32	0.632
Hypertension, n (%)	47 (81.03%)	45 (77.59%)	41 (67.21%)	0.222
SBP (mmHg)	128.0 (120.0–136.3)	129.0 (120.5–140.0)	125.00 (114.5–136.5)	0.269
DBP (mmHg)	77.50 (70.0–85.3)	73.0 (67.0–80.0)	75.0 (69.0–80.0)	0.222
Diabetes, n (%)	29 (50.00%)	27 (46.55%)	13 (21.31%)	0.002b
Fasting glucose (mmol/L)	5.90 (5.20–6.50)	5.90 (5.00–6.50)	5.90 (4.85–7.10)	0.957
Smoking, n (%)	21 (36.21%)	25 (43.10%)	27 (44.26%)	0.683
Alcohol, n (%)	12 (20.69%)	20 (34.48%)	21 (34.43%)	0.193
BMI (kg/m2)	27.10 (24.10–28.35)	25.65 (24.00–27.36)	26.00 (23.82–28.31)	0.197
TC (mmol/L)	3.81 (3.37–4.86)	3.95 (3.36–4.70)	4.10 (3.48–4.71)	0.950
TG (mmol/L)	1.35 (1.02–1.75)	1.50 (1.06–1.85)	1.25 (0.80–2.28)	0.438
HDL-c (mmol/L)	1.02 (0.87–1.27)	1.06 (0.88–1.26)	1.14 (0.88–1.38)	0.251
LDL-c (mmol/L)	2.09 (1.75–2.79)	2.16 (1.85–2.74)	2.16 (1.72–2.56)	0.819
ALT (U/L)	20.00 (13–28.25)	18.00 (15.00–24.25)	24.00 (17.00–28.00)	0.086
BUN (mmol/L)	5.80 (4.86–6.85)	5.35 (4.36–6.15)	5.13 (4.34–6.20)	0.093
Creatinine (µmol/L)	79.80 (69.23–90.10)	76.00 (67.00–86.44)	74.00 (66.05–89.25)	0.463

Plasma s-elastin and elastase (Table 2) were significantly higher in the CAE group than in the other two groups. Circulating inflammation-related indicators showed no significant differences between the CAE group and the other two groups. For the location of ectasia, CAG indicated that the most commonly involved vessel was the right coronary artery (53.45%).

**Table 2:** The inflammation indicators and coagulation function for all patients.

	CAE (n = 58)	CHD (n =5 8)	Relatively normal* (n = 61)	P
s-elastin (ng/mL)	17.48 (13.02–25.85)	13.01 (11.91–13.38)	11.95 (9.94–13.02)	0.000a
Elastase (ng/mL)	32.56 (26.43–35.66)	25.59 (20.33–28.77)	24.38 (20.57–28.54)	0.000b
hs-CRP, mg/L	1.42 (0.67–3.66)	1.35 (0.50–3.10)	1.29 (0.43–2.44)	0.248
Leukocytes (103/μL)	6.33 (5.24–7.34)	6.38 (5.21–8.05)	6.49 (5.43–7.28)	0.732
Neutrophils (103/μL)	6.30 ± 1.32	6.74 ± 1.96	6.51 ± 1.46	0.338
Monocytes (103/μL)	0.37 (0.29–0.48)	0.38 (0.32–0.46)	0.39 (0.31–0.47)	0.904
Lymphocytes (103/μL)	1.88 ± 0.56	1.79 ± 0.54	1.80 ± 0.52	0.619
N/L ratio	2.25 (1.59–2.74)	2.52 (1.77–2.98)	2.28 (1.61–2.95)	0.287
Ectatic vessels				
LM, n (%)	16 (27.59%)			
LAD, n (%)	27 (46.55%)			
LCX, n (%)	26 (44.83%)			
RCA, n (%)	33 (53.45%)			

Figure illustrates the ROC curve for s-elastin and elastase and presents the correlation analysis between them. The area under curve (AUC) with 95% confidence interval (CI) for s-elastin was 0.786 (0.714–0.858, P = 0.000). For elastase, the AUC with 95% CI was 0.775 (0.702–0.847, P = 0.000). The Pearson correlation analysis revealed that s-elastin was significantly correlated with elastase.

**Figure:**
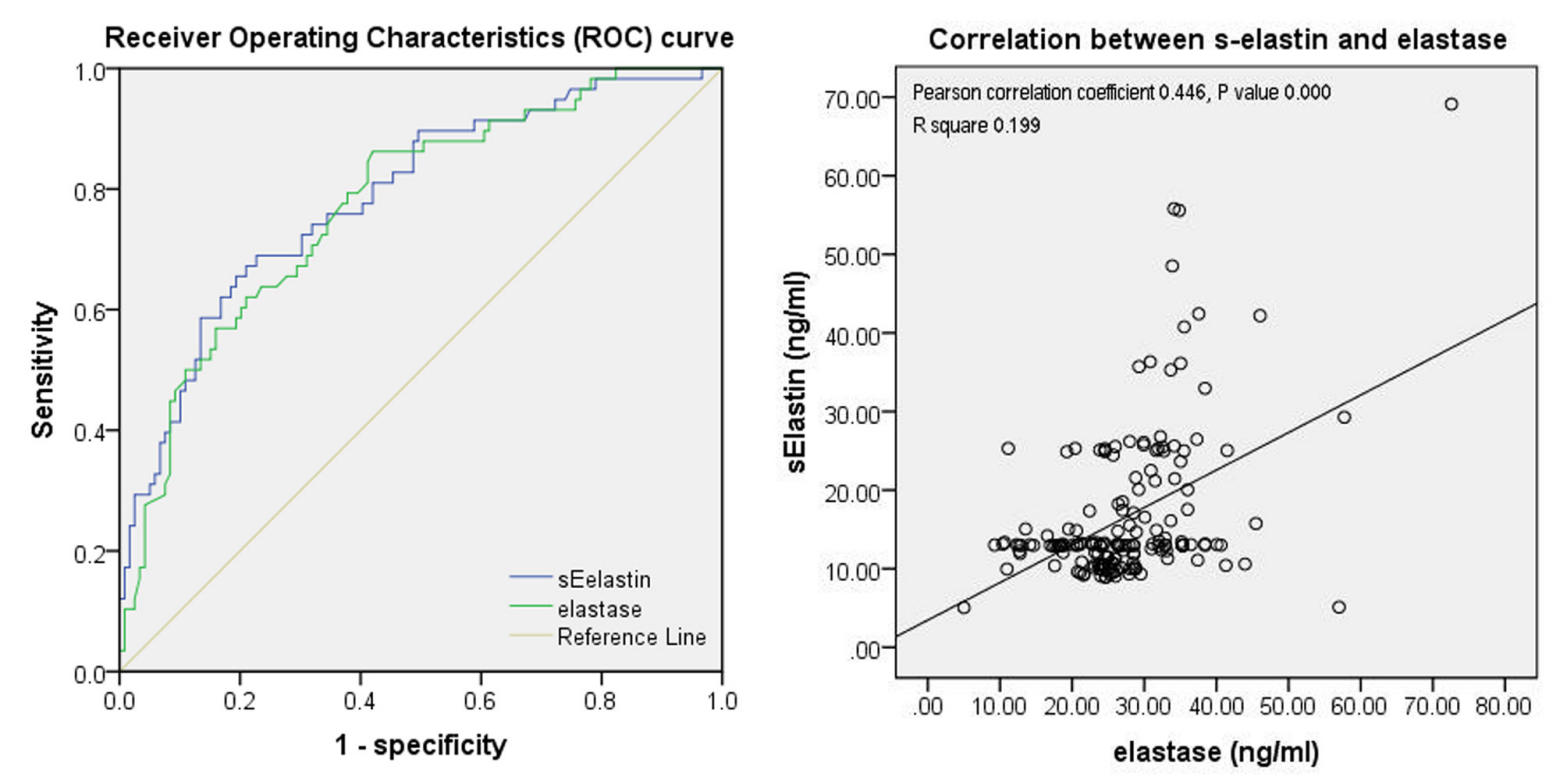
ROC curves for elastase and s-elastin.

Table 3 presents the evaluation of the diagnosis method for CAE with s-elastin and elastase. For s-elastin and elastase, the best cut-off values were 13.148 ng/mL and 25.549 ng/mL; the values for sensitivity were 0.690 and 0.773; and the values for specificity were 0.862 and 0.571, respectively. Further analyses showed that the combination of s-elastin and elastase in series (one of the two higher than its cut-off value as the diagnosis standard) was suitable for screening CAE with a better sensitivity, whereas their combination in parallel (both higher than their cut-off values as the diagnosis standard) was suitable for identifying CAE with a better specificity.

**Table 3:** The diagnostic evaluations of elastase and s-elastin for patients with CAE.

Items		s-elastina		Elastaseb		Combined in parallelc		Combinedin seriesd	
		-	+	-	+	-	+	-	+
CAGe	-	90	29	68	51	103	16	55	64
	+	18	40	8	50	22	36	4	54
Positive prediction		57.97%		49.50%		69.23%		45.76%	
Negative prediction		83.33%		89.47%		82.40%		93.22%	
Positive likelihood ratio		3.04		2.01		4.62		1.73	
Negative likelihood ratio		0.40		0.24		0.44		0.15	
Sensitivity		0.690		0.862		0.621		0.931	
Specificity		0.773		0.571		0.866		0.462	
Yoden index		1.463		1.433		1.486		1.393	
Diagnostic index		0.463		0.433		0.486		0.393	
Coincidence rate		73.45%		66.67%		78.53%		61.58%	
Missed diagnosis rate		31.00%		13.80%		37.93%		6.90%	
Misdiagnosis rate		22.70%		42.90%		13.45%		53.78%	
Kappa value		0.425		0.364		0.499		0.311	
Kappa test		0.000		0.000		0.000		0.000	

When the patients were stratified into the four Markis subgroups (Table 4), no intergroup significant differences were noted in plasma s-elastin and elastase concentrations.

**Table 4:** The s-elastin and elastase in different Markis types.

	Markis I (n = 10)	Markis II (n = 14)	Markis III (n = 26)	Markis IV (n = 8)	P
Age, years	66.20 ± 8.40	61.14 ± 9.94	65.08 ± 10.57	69.25 ± 10.71	0.320
Sex, male (%)	6 (60.00%)	10 (71.43%)	15 (57.69%)	4 (50.00%)	0.509
BMI (kg/m2)	25.53 ± 4.80	28.31 ± 2.98	26.83 ± 3.10	25.93 ± 1.51	0.185
Hypertension, n (%)	10 (100.00%)	12 (85.71%)	20 (76.92%)	5 (62.50%)	0.202
SBP (mmHg)	133.5 (128.8–141.3)	121.0 (116.3–138.8)	127.00 (119.8–137.3)	127.5 (125.0–132.0)	3.456
DBP (mmHg)	136.3 ± 17.0	127.3 ± 15.6	129.2 ± 18.7	128.0 ± 5.0	0.064
Diabetes, n (%)	5 (50.00%)	9 (64.29%)	9 (34.62%)	6 (75.00%)	0.133
TC, (mmol/L)	4.00 (3.57–5.28)	4.08 (3.40–4.75)	3.64 (3.24–4.41)	4.35 (3.75–5.37)	3.328
TG, (mmol/L)	1.73 (1.22–3.16)	1.42 (0.89–1.96)	1.33 (1.06–1.54)	1.32 (0.96–1.80)	2.868
HDL-c, (mmol/L)	1.01 (0.82–1.51)	1.02 (0.79–1.30)	1.01 (0.87–1.20)	1.09 (0.96–1.31)	0.995
LDL-c, (mmol/L)	2.29 ± 0.59	2.31 ± 0.65	2.29 ± 0.87	2.69 ± 0.98	0.632
Smoking, n (%)	5 (50.00%)	6 (42.86%)	6 (23.08%)	4 (50.00%)	0.268
Alcohol, n (%)	3 (30.00%)	2 (14.29%)	4 (15.38%)	3 (37.50%)	0.469
N/L ratio	2.07 (1.66–2.71)	2.39 (1.67–2.87)	2.30 (1.51–2.62)	2.00 (1.41–3.13)	0.559
hs-CRP	0.71 (0.36–8.10)	2.20 (0.65–3.96)	2.03 (0.65–4.56)	1.14 (0.77–1.42)	1.407
ALT (U/L)	14.00 (9.75–20.75)	23.00 (19.25–30.50)	21.50 (13.00–33.75)	16.00 (14.00–20.75)	5.497
BUN (mmol/L)	5.51 (4.18–6.78)	5.59 (4.18–7.43)	5.92 (4.92–6.57)	5.80 (5.36–6.39)	0.228
Creatinine (µmol/L)	83.15 (67.75–102.50)	80.30 (71.33–90.45)	78.50 (66.33–94.30)	72.75 (65.30–77.95)	1.702
s-elastin (ng/mL)	13.90 (11.11–25.58)	25.03 (14.61–28.47)	17.26 (13.02–27.41)	17.16 (12.96–25.38)	2.515
Elastase (ng/mL)	27.10 (25.39–35.46)	33.27 (29.43–35.66)	33.38 (27.71–37.77)	28.96 (24.89–35.01)	3.250

## 4. Discussion

In this study, the CAE, CHD, and relatively-normal-coronary groups were balanced with most of the baseline characters, and 95% of the patients with CAE had concomitant CHD. On the basis of the detection of plasma s-elastin and elastase concentrations in patients with CAE, the possible diagnostic value of applying these two indicators was explored: 1) Plasma s-elastin and elastase were significantly increased in the CAE group compared to the CHD and relatively-normal-coronary groups. 2) The ROC curve showed that s-elastin of 13.148 ng/mL and elastase of 25.549 ng/mL were the best cut-off values for CAE diagnosis with acceptable values of sensitivity and specificity. 3) The combination of s-elastin and elastase in series (one of the two was higher than its cut-off value) was suitable for screening CAE with a better sensitivity, while the combination of s-elastin and elastase in parallel (both were higher than their cut-off values) was suitable for identifying CAE with a better specificity. 4) There was no significant relationship among different Markis types for s-elastin and elastase.

This research indicated that detecting peripheral elastase and s-elastin is a promising method for screening CAE. The characteristic pathological change for CAE is the destruction of elastic fibres in the coronary arteries. Coronary arteries normally comprise tens of layers of units composed of vascular smooth muscle cells and fibrous layers, which are mainly composed of elastic fibres (collagen types I and III) [4]. The synthesis of elastin in vessels ceases in adulthood [12], so its degradation or destruction is an irreversible phenomenon. Once degraded, the elastic fibres are broken down into s-elastin and polypeptide fragments, and then released into the peripheral circulation [13]. Elastin fibres are the main targets of neutrophil serine proteases, including elastase, proteinase 3, and cathepsin G [14]. Thus, theoretically, increased elastase and s-elastin concentrations in the peripheral circulation might be indicators of coronary artery destruction in patients with CAE. However, they can also be elevated in patients with aneurysms in other vessels because there might be a common underlying pathological process for various aneurysmal diseases. Therefore, these findings should be correlated with the patient’s atypical angina symptoms with ischaemic changes to rule out other aneurysmal diseases.

Appropriate diagnostic biomarkers for CAE have been lacking because the current understanding of its pathogenesis is relatively limited due its low incidence rate and because the pathological specimens are not easy to acquire. The most critical discovery was that CAE is a type of systemic inflammatory disease [15,16]. Moreover, inflammatory cells infiltrated the coronary wall, and many peripheral inflammatory molecules and cytokines were also disordered, such as high-sensitivity C-reactive protein (hs-CRP), neutrophil-to-lymphocyte ratio, red cell distribution width, interleukin (IL)-4, IL-6, IL-2, tumour necrosis factor, E-selectin, P-selectin, intercellular cell adhesion molecule-1, and vascular cell adhesion molecule 1, as well as the oxidant-antioxidant indicators [15,16]. However, most of those indicators lack sensitivity or specificity when used for CAE diagnosis, and it is challenging to connect them to the pathological process of CAE. This study demonstrated that plasma s-elastin and elastase concentrations had better sensitivity and specificity for diagnosing CAE; this also makes sense from the viewpoint of pathogenesis. A combination of s-elastin and elastase concentrations in series increased the diagnostic sensitivity and in parallel, provided increased specificity. Detection and quantification of s-elastin and elastase is fast, easy, and inexpensive. In patients with atypical angina, clinicians might consider routine examination of s-elastin and elastase for early and convenient detection of coronary dilatation.

## 5. Limitations

This study has some limitations. First, this study had a small sample size and required to be testified by large simple size studies and by prospective studies. Second, follow-up observations are needed to clarify the prognoses, survival rates, and terminal events for each group. Third, the control group was defined as coronary artery stenosis <20% but not normal according to current consensus.

## 6. Conclusion

Circulating s-elastin and elastase are promising biomarkers for assisting CAE diagnosis with acceptable values of sensitivity and specificity, and the auxiliary diagnosis method by these two biomarkers makes sense theoretically and pathologically. Further, the combination of s-elastin and elastase in series (one of the two was higher than its cut-off value) was with a better sensitivity for screening CAE, while in parallel (both the two were higher than their cut-off values) was with a better specificity for identifying CAE.

## Informed consent

This research was approved by the Ethics Committee of the Beijing Friendship Hospital and was in line with the Helsinki Declaration. All subjects signed informed consent paperwork (YYBB-B01-01-R02-V4.0). 
